# SIMIND Monte Carlo simulation of a single photon emission CT

**DOI:** 10.4103/0971-6203.55967

**Published:** 2010

**Authors:** M. T. Bahreyni Toossi, J. Pirayesh Islamian, M. Momennezhad, M. Ljungberg, S. H. Naseri

**Affiliations:** Medical Physics Research Center, Mashhad University of Medical Sciences, Iran; 1 Medical Physics Department, Faculty of Medicine, Mashhad University of Medical Sciences, Mashhad, Iran; 2 Nuclear Medicine Department, Faculty of Medicine, Mashhad University of Medical Sciences, Mashhad, Iran; 3 Medical Radiation Physics Department, Clinical Sciences-Lund, Lund University, Lund, Sweden

**Keywords:** Emission tomography, Monte Carlo simulation, SIMIND, SPECT, ^99m^Tc imaging

## Abstract

In this study, we simulated a Siemens E.CAM SPECT system using SIMIND Monte Carlo program to acquire its experimental characterization in terms of energy resolution, sensitivity, spatial resolution and imaging of phantoms using ^99m^Tc. The experimental and simulation data for SPECT imaging was acquired from a point source and Jaszczak phantom. Verification of the simulation was done by comparing two sets of images and related data obtained from the actual and simulated systems. Image quality was assessed by comparing image contrast and resolution. Simulated and measured energy spectra (with or without a collimator) and spatial resolution from point sources in air were compared. The resulted energy spectra present similar peaks for the gamma energy of ^99m^Tc at 140 KeV. FWHM for the simulation calculated to 14.01 KeV and 13.80 KeV for experimental data, corresponding to energy resolution of 10.01 and 9.86% compared to defined 9.9% for both systems, respectively. Sensitivities of the real and virtual gamma cameras were calculated to 85.11 and 85.39 cps/MBq, respectively. The energy spectra of both simulated and real gamma cameras were matched. Images obtained from Jaszczak phantom, experimentally and by simulation, showed similarity in contrast and resolution. SIMIND Monte Carlo could successfully simulate the Siemens E.CAM gamma camera. The results validate the use of the simulated system for further investigation, including modification, planning, and developing a SPECT system to improve the quality of images.

## Introduction

Recent developments in nuclear medicine instrumentation and multiple-processor parallel processing systems have created a need for Monte Carlo simulation opportunities in nuclear medicine imaging. Since nuclear medicine imaging deals with random phenomena such as radioactive decay, emission of radiation energy through photons and particles, and the detection of these quanta and particles in various material, Monte Carlo simulations are nowadays employed as an essential tool in nuclear medicine imaging, both in single-photon emission computed tomography (SPECT) and positron emission tomography (PET).[1–3] One of the aims of a medical physicist, involved in nuclear medical imaging research, is to optimize the design of imaging systems and improve qualitative and quantitative accuracy of reconstructed images. Several factors affect image quality and accuracy of the data obtained from a nuclear medicine scan. These include: physical properties of detectors, collimator and gantry design, attenuation and scatter compensation and reconstruction algorithms.[4,5] Integrated improvement in these parameters with current tracers and sensitive and specific tracers under development will provide major advantages to the general nuclear medicine clinician and research investigator. Mathematical modeling is necessary for assessment of various parameters in nuclear medical imaging systems since no analytical solution is possible when solving the transport equation describing the interaction of photons with nonuniformly attenuating body structures and complex detector geometries. One of the most frequent applications of Monte Carlo methods in nuclear medicine imaging is detector modeling with three main purposes: to study interactions within the radiation sensor for each photon and thus correct for sources of image degradation; evaluate techniques of image treatment to quantify the effects of dispersion and attenuation, and to optimize reconstruction algorithms; and patient dosimetry calculations. To improve quality, some authors have suggested improving the detection system itself, especially the collimator.[6–8] Monte Carlo simulation appears to be the best solution to model gamma-camera behavior,[9–16] although the long calculation time implied has often limited its application. A detailed description of the general principles of the Monte Carlo method is given in a number of publications.[17–20]

A number of Monte Carlo simulation codes (e.g. EGS4, MCNP4, SimSET, Geant4, Gate, and SIMIND) applicable to nuclear medicine have been developed and described in literature.[6–8]

The design of SPECT and PET systems using the Monte Carlo method has received considerable attention and such investigations resulted in several applications.[21–22] The study used the SIMIND Monte Carlo simulation program, which is well established for SPECT with low-energy photons, for photonic physics and other applications. This program was originally designed for the calibration of whole-body counters, but soon evolved to simulate scintillation cameras.[1,23–24] It is now available in Fortran-90 and can be freely downloaded from http://www.radfys.lu.se/simind and run on major computer platforms including PCs.[25] The SIMIND program actually consists of two programs, CHANGE, which defines the parameters, and SIMIND, which performs the actual simulation. The program can simulate nonuniform attenuation from voxel-based phantoms and includes several types of variance reduction techniques. Transmission SPECT images can also be simulated. For particular projects, the user can write scoring routines linked to the main code. The program has been modified to allow it to be run on parallel computers using the MPE command language. The use of SIMIND is well documented.[2]

In this article, we present the simulation of a Siemens E.CAM gamma camera,[26] used in clinical practice in our center.

## Materials and Methods

### Gamma Camera

The Siemens E.CAM gamma camera was modeled using SIMIND Monte Carlo program. The camera consists of a removable low energy high resolution (LEHR) collimator, a NaI (Tl) scintillation crystal, a light guide and an array of photomultiplier tubes (PMTs). The parameters of LEHR collimator, used for low energy sources such as ^99m^Tc, for experiment and simulation were as follows: parallel hexagonal holes with cells of 1.11 mm diameter, 2.405 cm height, and 0.16 mm septal thickness. The NaI (Tl) crystal specifications are as follows: planar, 9.5 mm in thickness, 59.1 × 44.5 cm^2^ in area, light yield 40k photons /MeV, and a peak emission spectrum at 415 nm.[27,28] Generated light in the crystal is collected by a matrix composed of 59 PMTs, 53 with 7.6 cm and 6 with 5.1 cm in diameter. The photocathode is a bialkali type with quantum efficiency of approximately 30% for the wavelength of maximum NaI (Tl) emission.[29] A light guide ensures a good optical coupling between the scintillating crystal and PMTs. The SIMIND Monte Carlo program was utilized to simulate the aforementioned structures. When ^99m^Tc is used, various structures attached to back of the crystal contribute to backscattering of the emitted photons. To assess the effect of these parts, a single 6 cm slab of Pyrex[30] was substituted and simulated.

The scintillation process was simulated for generation of optical photons within the NaI (Tl) crystal. An isotropic source emitting 140 keV photons was assigned to ^99m^Tc. To obtain the output spectra of the simulated gamma camera a ^99m^Tc point source was simulated for the following geometries and activities:

- 3.7 MBq, without collimator, positioned at 0, 10, 15, 20, and 25cm from the detector surface.
- 37 MBq, with collimator, and at the same distances as in (a).

The experimental output spectra of the real gamma camera were acquired for exactly the same conditions as applied to the simulated system. Acquired data representing the system spectra was stored in digital format. An excel program was employed to display the energy spectra.

In this work we have studied the following properties of the gamma camera:[31–37] energy resolution, spatial resolution and image contrast. An energy window was centered on the ^99m^Tc photo peak (130 - 151 keV).[36] The images were reconstructed in matrices of 128× 128 pixels, with a pixel size of 0.39 mm.

### Energy Resolution

Energy resolution of the gamma camera was measured with a ^99m^Tc (3.7MBq) point source, positioned at the center of the field of view (FOV), 25 cm from the crystal surface. The energy spectrum was acquired for 10^7^ photons / projection. The simulated and experimental energy spectra were obtained with and without a low energy high resolution collimator for a source-detector distance of 25 cm.

### Spatial Resolution

Spatial resolution of the real and simulated gamma camera were determined by placing a ^99m^Tc (1.9MBq) source (1.5 mm in diameter) at the center of the FOV. A study of the SPECT reconstructed spatial resolution was also carried out both experimentally and by SIMIND simulation. SPECT projections of a Jaszczak Deluxe Phantom[39] along the axis of rotation were acquired. The phantom was uniformly filled in with 370MBq ^99m^Tc, and positioned 15 cm from the collimator surface. The projections were obtained in the 15% window (130 - 151 keV). Spatial resolution (in mm) was obtained by the smallest visible and recognizable rods.

### Sensitivity

Sensitivity of the gamma camera is determined by taking ratio of the detected counts per second in the selected energy window per unit activity in the source (cps/MBq). The system sensitivity was experimentally measured and estimated by simulation for the point source with the condition specified in section 2. The measured sensitivity was obtained for a 3.7 MBq source counted over a period of 60 seconds, whereas the corresponding simulation was performed for 10 million photons generated for the same source position. Decay time and background radiation were taken into consideration. Finally, the response of the E-CAM in extrinsic mode (i.e., with the collimator) was studied for a point source placed 25 cm from the detector.

### Imaging Evaluation

The Jaszczak Deluxe phantom was also employed to evaluate the quality measurement of images obtained experimentally and by simulation.[38–39] The SPECT parameters were the same as already described in section 3, also same configuration was applied to SIMIND simulation of the phantom. The acquisition parameters were identical for experiments and simulations: 128×128 matrix, 128 views, 1.23 zoom factor, 3.9 mm pixel size, and with approximately 1 million counts / projection resulting in 128 million total counts.

## Results and Discussion

### Energy Resolution

**Figure 1:**
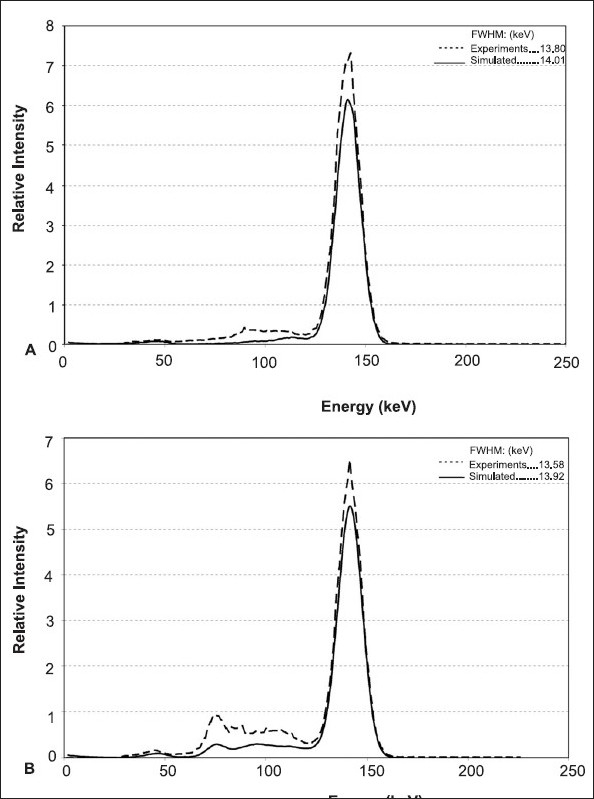
Energy Spectra of the Siemen's E-Cam modeling, for a ^99m^Tc point source at 25 cm from the NaI(Tl) detector, (A) without and (B) with a LEHR collimator and one million photons. Related SIMIND simulated spectra (solid) and Experimental energy spectra (dashed) are presented

Figure 1 shows the energy spectra produced by the real and simulated gamma cameras for a ^99m^Tc point source, positioned 25 cm from the detector surface. Some minor differences may be observed between the simulated and the experimental energy spectra, the most striking being that the experimental spectrum presents a wider peak which may be explained by the superimposition of the energy peaks corresponding to the X-ray escape of ^53^I present in the NaI (Tl) crystal (~110 keV), the ^99m^Tc photo peak (140 keV) and the sum of 140 keV with the x-ray energy of ^99m^Tc (total ~160 keV),[40] which cannot be separated by the detector. However, the results obtained for the spectrum peak at 140 keV is satisfactory.

The calculated FWHMs for 140 keV photo peak were equal to 14.01 keV and 13.80 keV for simulated and experimental normalized energy spectra, respectively. The corresponding relative energy resolution, with and without a LEHR collimator, obtained for simulated and experimentally acquired spectra are 9.94%, 9.61% and 10.01%, 9.86%, respectively. Contributions of Compton and photoelectric interactions in the whole spectra (simulated) and in the selected window are given in Table 1. There is a good agreement between the results obtained from simulations and experiments. However, there were some differences between simulated results obtained for the FWHMs that were constant over the range of source-collimator selected distances. The differences are due to the fact that the size of point source defined by the SIMIND is negligible compared to the 1.5 mm diameter of the source used in experimental measurements. Our measured and computed relative energy resolution (intrinsic) compare very well with the 9.9% value claimed by the manufacturer.[28]

**Table 1:** Results from energy spectrum of a ^99m^Tc point pource simulated with SIMIND

*Interaction type*	*Relative contribution*	% *(1SD)*
Compton Area in Spectrum	48.56	1.72
Photoelectric Area in	316	0.53
Spectrum		
Pileup Area in Spectrum	13	3.69
Fraction Photo in Window	0.9998	0.53
Fraction Compton in Window	0.0002	68.50

### Spatial Resolution

Spatial resolution for a source to collimator distance of 10 cm was found to be 8.4 plus/minus 0.1 mm and 7.8 plus/minus 0.1mm for the actual and simulated systems, respectively. The simulated and experimental spatial resolutions were closely the value provided by manufacturer for a point source at this distance is 7.8 mm,[26] which is in good agreement with our experimental value.

**Figure 2:**
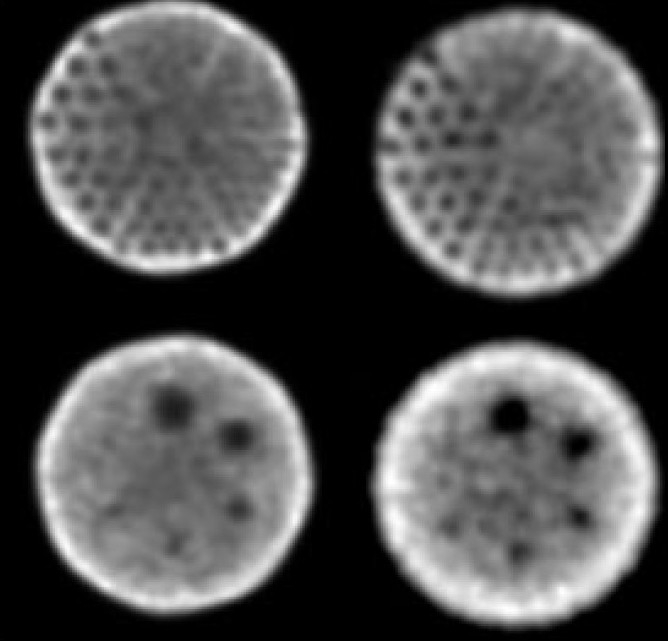
Images of experimental (right) and simulated (left) SPECT Jaszczak Deluxe phantom acquisition, consisting of six cold spheres and 148 rods, filled with 370MBq ^99m^Tc. The acquisition parameters were 128 × 128 matrix, 128 views, 1.23 zoom factor, 3.9-mm pixel size, 30 second acquisition time per view using a dual-head camera (E.CAM TM; Siemens Medical Systems). Images were reconstructed by filtered back projection reconstruction using a Butterworth filter with a cut-off frequency of 0.5. The cold rod diameters are from top right clockwise: 4.8, 6.4, 7.9, 9.5, 11.1 and 12.7 mm. Approximately 128 Mcounts were recorded for simulations

**Figure 3:**
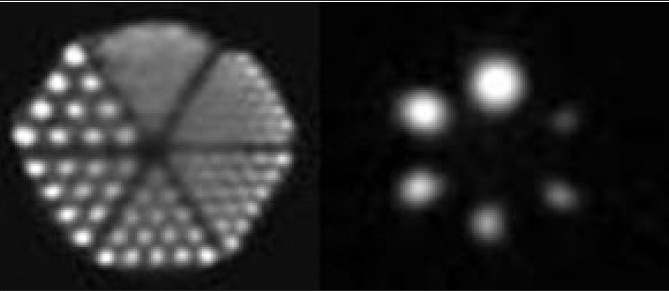
Images of Simulated SPECT Jaszczak Deluxe phantom acquisition, consisting of 148 rods and six hot spheres with different diameters (9.5, 12.7, 15.9, 19.1, 25.4, and 31.8 mm) filled with 10mCi ^99m^Tc. The acquisition parameters were 128 × 128 matrix, 128 views, 1.23 zoom factor, 3.9-mm pixel size, approximately one million counts per view, 128 million total counts. Images reconstructed by filtered back projection reconstruction using a Butterworth filter with a cut-off frequency of 0.5

To evaluate the spatial resolution of reconstructed SPECT of a Jaszczak phantom, the images were obtained by experiment and modeling. Figure 2 shows corresponding images of the cold rods and spheres in the phantoms, prepared by simulation and by experiment. The best results were obtained for the simulated hot rods and spheres Figure 3. The qualities of the produced images were compared in terms of image contrast and spatial resolution.[41] From Figures 2 and 3 it is evident that reconstructed spatial resolution of both SPECTs is nearly equal to 9.5mm. Image contrasts for the six spheres were calculated by equations (1) and (2) and presented in Table 2.

**Table 2:** Results for calculated contrast of Jaszczak phantom spheres from reconstructed SPECT acquisitions

*Condition*	*Spheres size (mm)*					
	
	*31.8*	*25.4*	*19.1*	*15.9*	*12.7*	*9.5*
Cold experiment	0.774	0.627	0.575	0.372	0.191	0.132
Cold simulated	0.661±0.003	0.527±0.007	0.487±0.007	0.400	0.23±0.003	0.2±0.004
Hot simulated	0.92±0.002	0.91±0.002	0.88±0.008	0.81±0.009	0.76±0.001	0.56±0.001

Eq. 1 $${\text{Contrast}}_{\text{H}}={\text{N}}_{\text{sp}.}/{\text{N}}_{\text{b}.}$$

Eq. 2 $${\mathrm{Contrast}}_{C}=1-(M_{\mathrm{sp}}./M_{\mathrm{cy}.})$$

The N_sp_ and N_b_ are the mean pixel values of the hot spheres and background, respectively. M_sp_ and M_cy_ correspond to the minimum pixel value in cold spheres and the maximum pixel values in the phantom cylinder, respectively.

From Table 2 it can be seen that contrast decreased with decreasing sphere diameter, as would be expected. The calculated contrasts for cold and hot simulated spheres with the diameter larger than 15.9 mm have a remarkable value compared to the diameter of 9.5 mm. Similar results were also seen for the experiment with an apparent similarity for sphere sizes above the diameter of 15.9 mm. However, there was no significant change in contrast for the 12.7- and 9.5-mm spheres. Therefore, the obtained contrast similarity for cold spheres, as an imaging parameter, is another evident for verification of the simulation.

### Sensitivity

Sensitivities of the actual and simulated gamma cameras were equal to 85.11 and 85.39 cps/MBq. Although these figures are not significantly different, but the small difference is due to acquisition dead time and signal overlap in the detector, which are not taken into account for the latter system. In Figure 1, the backscattering pattern obtained for the simulated and real systems (Compton region) are very similar, confirming that the six cm Pyrex slab is a justifiable substitute for the whole structures attached to the back of NaI (Tl) crystal in the simulated system.

## Conclusion

Comparison of acquired results by simulation and experiment is good evidence that SIMIND Monte Carlo simulation is capable of simulating the Siemens E.CAM gamma camera successfully. Rodrigues *et al*,[40] have also modeled a similar gamma camera with GATE.[42] The two codes are slightly different. Physical and geometrical parameters applied in these two studies are not exactly identical. However, taking into account the effect of these variations, the minor differences between two sets of results are negligible.

In summary, comparing the results acquired experimentally to those obtained by Monte Carlo simulation validate the simulated SPECT system. This step is a prerequisite for using the simulated system for complex studies on optimization of the performance characteristics, and finally improving the quality of SPECT produced images.
